# Temperature Responsive Copolymers Films of Polyether and Bio-Based Polyamide Loaded with Imidazolium Ionic Liquids for Smart Packaging Applications

**DOI:** 10.3390/polym15051147

**Published:** 2023-02-24

**Authors:** Daniela C. Zampino, Gabriele Clarizia, Paola Bernardo

**Affiliations:** 1 Institute of Polymers, Composites and Biomaterials (IPCB-CNR), Via P. Gaifami 18, 95126 Catania, Italy; 2 Institute on Membrane Technology (ITM-CNR), Via P. Bucci 17/C, 87036 Rende, Italy

**Keywords:** Imidazolium Ionic Liquids, temperature responsive materials, gas separation, polyether block amide

## Abstract

Temperature-responsive materials are highly interesting for temperature-triggered applications such as drug delivery and smart packaging. Imidazolium Ionic Liquids (ILs), with a long side chain on the cation and a melting temperature of around 50 °C, were synthetized and loaded at moderate amounts (up to 20 wt%) within copolymers of polyether and a bio-based polyamide via solution casting. The resulting films were analyzed to assess their structural and thermal properties, and the gas permeation changes due to their temperature-responsive behavior. The splitting of FT-IR signals is evident, and, in the thermal analysis, a shift in the glass transition temperature (*T*g) for the soft block in the host matrix towards higher values upon the addition of both ILs is also observed. The composite films show a temperature-dependent permeation with a step change corresponding to the solid–liquid phase change in the ILs. Thus, the prepared polymer gel/ILs composite membranes provide the possibility of modulating the transport properties of the polymer matrix simply by playing with temperature. The permeation of all the investigated gases obeys an Arrhenius-type law. A specific permeation behavior, depending on the heating–cooling cycle sequence, can be observed for carbon dioxide. The obtained results indicate the potential interest of the developed nanocomposites as CO_2_ valves for smart packaging applications.

## 1. Introduction

Smart polymeric materials, capable of reacting to small changes in the environment, are actively studied in the fields of nanoscience, nanotechnology and biomedical engineering for numerous applications [1]. Temperature-responsive polymers are of practical interest since their physical properties can be varied by changes in the external temperature [2]. As this is an easily measurable and controllable parameter, the thermal stimuli can be induced externally and thus in a non-invasive manner, as required in biomedical applications.

The loading of temperature-responsive materials into a polymeric matrix to prepare packaging systems is a strategy to regulate the permeation of gases or water vapor in response to temperature changes occurring on the external atmosphere. Among various temperature-responsive materials, Ionic Liquids (ILs) are salts with a low vapor pressure. By playing with the chemical structure of the ionic constituents, ILs can be solid at room conditions and thus can be adopted to induce a discontinuous thermal behavior in a polymer matrix, depending on their melting temperature. In particular, long-chain alkylimidazolium ILs (e.g., [C_16_MIM]Br and [C_16_MMIM]Br) have high heat capacity and high storage density [3].

Pebax^®^ are block copolymers in which linear chains of hard polyamide (PA) are covalently linked to soft polyether (PE) blocks via ester groups [4]. The soft blocks, owing to dipole–dipole interactions, are more flexible [4] and provide sustained gas permeability, while the hard PA segments guarantee mechanical stability. Different properties are available in these copolymers, depending on the PE and PA types and on their relative ratio. The biocompatibility enables the application of Pebax in the medical field (e.g., for virus-proof surgical sheeting or for short-term implants in humans) [5]. Flexible packaging is another application of these thermoplastic elastomers, as well as in selective separation of membranes. Indeed, sorption and permeation tests have evidenced the good affinity of the PE blocks in Pebax for the polar CO_2_ gas molecules [6]. Accordingly, several studies have investigated the use of Pebax as membrane material, alone or including ILs [7,8,9], non-ionic surfactants [10,11], nanoparticles [12,13], or combined with both ILs and nanofillers [14,15].

In the present study, blends of the commercial copolymer Pebax^®^Rnew and two long-chain alkylimidazolium ILs ((HdmimDMSIP), with a C16 side chain on the Imidazolium ring in the cation and (OOMmimPF_6_) an ether C9 chain on the Imidazole-based cation), were developed as flexible films. Pebax^®^Rnew is based on Polyamide 11 (PA11), an aliphatic semi-crystalline homopolymer obtained from renewable raw materials. Its physical and mechanical properties (e.g., low water absorption, high tensile strength and stiffness) are mainly due to the hydrogen bonding between adjacent chains of the highly polar amide (–CONH–) groups [16]. Dense films were prepared by solution casting, immobilizing the synthetized IL salts within the polymer matrix, and using ethanol as solvent.

These novel Pebax/ILs composites as self–supported membranes were investigated regarding their structural and thermal properties, and the membranes were tested in gas separation, analyzing the effect of temperature on its potential applications in flexible packaging. Both of the selected ILs have a melting point of around 50 °C, thus, the prepared films were characterized in the same temperature range to study the effect of their solid–liquid transition on the permeation of gases. In particular, they have both an Imidazolium-based cation and Imidazolium ILs, which are more thermally stable than tetraalkyl ammonium-, piperidinium- and pyridinium-based ILs, independent of the anion [17]. The attractive features of these blends are represented by the use of a flexible and bio-derived thermoplastic copolymer of a green solvent [18], used for material processing and in thermally stable additives (Imidazolium ILs) that can display various phase change temperatures on the basis of the ionic constituents.

## 2. Experimental

### 2.1. Materials

1-methylimidazole, 1,3-dimethyl 5-sulfoisophthalate sodium salt, 1-bromohexadecane, chloromethyl octyl ether, sodium hexafluorophosphate, tetrahydrofuran, dichloromethane, hexane, ethyl acetate, dimethyl sulfoxide-d6 and trans-2-[3-(4-tert-butylphenyl)-2-methyl-2-propenylidene]malononitrile, supplied by Sigma Aldrich (Milan, Italy), were used in the ILs synthesis. Ethanol absolute from VWR (Milan, Italy) was applied as a solvent in the membrane prepared. Chemicals were utilized as received.

The block copolymer Pebax^®^Rnew 25R53, based on a bio-polyamide 11 (PA11) and poly(tetramethylene oxide) (PTMO, in the range 79–83 wt% [19]), was received from Arkema (Milan, Italy).

Gases for permeation tests, including He, H_2_, O_2_, N_2_ and CO_2_, were purchased with a purity of 99.99+% from SAPIO (Monza, Italy).

### 2.2. Methods

#### 2.2.1. IL Synthesis and Characterization

**Figure 1:**
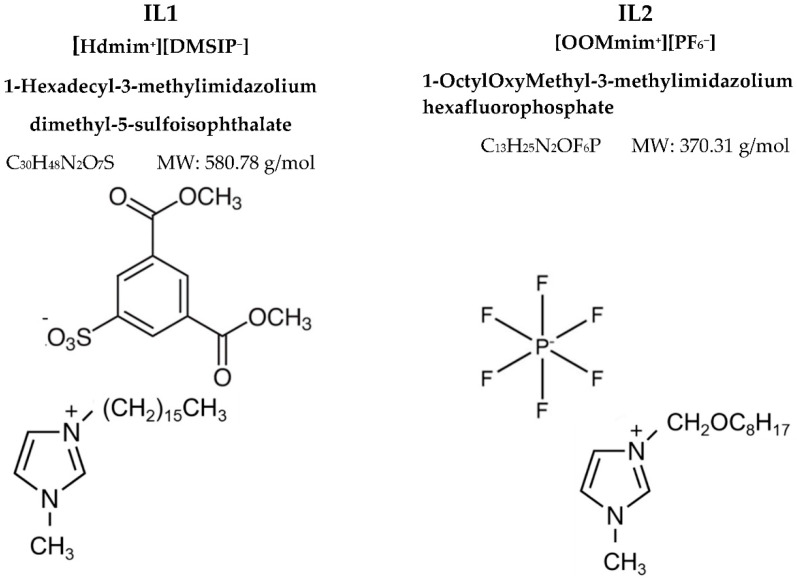
The chemical structures of the synthetized ILs.

The structure of the ILs is reported in Figure 1. The synthesis of the ILs and their characterization by ^1^H Nuclear Magnetic Resonance spectroscopy (^1^H NMR) and Matrix-Assisted Laser Desorption Time of Flight Mass Spectrometry (MALDI TOF MS) analyses was previously described [20,21]. Both ILs were synthetized according to a two-step method.

*Synthesis of 1-hexadecyl-3-methylimidazolium dimethyl-5-sulfoisophthalate (HdmimDMSIP)*

The first step was the production of 1-hexadecyl-3-methylimidazolium bromide (HdmimBr) by reacting 1-bromohexadecane (6.4 mL, 20 mmol) with a solution of 1-methylimidazole (1.56 mL, 20 mmol) in 4 mL of ethyl acetate. The reaction was carried out at 65 °C under stirring in an inert nitrogen atmosphere for 24 h. The produced 1-hexadecyl-3-methylimidazolium bromide (HdmimBr) was cooled down to room temperature and filtered. A final washing with the ethyl acetate solvent enabled the removal of unreacted starting materials, leaving a white powder that was dried at 40 °C for 24 h in a vacuum oven (yield 95.6%).

The second step was a metathesis reaction between the HdmimBr (20 mmol, 7.77 g) in 40 mL of dichloromethane (DCM) and a solution of 1,3-dimethyl 5-sulfoisophthalate sodium (NaDMSIP, 20.9 mmol, 6.03 g) in water (130 mL). The two solutions were placed in a separating funnel and intensely shaken (30–45 min), reaching a condition in which two clear phases were separated without any precipitate. The organic layer was recovered, dried over magnesium sulfate and filtered. A Rotavapor removed the residual solvent. The HdmimDMSIP product was washed using ethyl acetate and dried for 24 h in a vacuum oven at 40 °C (yield 92.0%).

*Synthesis of 1-octyloxymethyl-3-methylimidazolium hexafluorophosphate (OOMmimPF_6_)*

The first step was the production of 1-octyloxymethyl-3-methylimidazolium chloride (OOMmimCl), which was obtained by stirring a mixture of chloromethyl octyl ether (3.86 mL, 0.02 mol) and 1-methylimidazole (1.585 mL, 0.02 mol) under nitrogen. The reaction was carried out for 30 min at room temperature. The obtained OOMmimCl was purified by repeating hot hexane (at 50 °C) washings and was recovered by filtration.

The second step was a metathesis reaction between a water solution (10 mL) of OOMmimCl (0.02 mol) and a water solution (10 mL) of sodium hexafluorophosphate (NaPF6, 0.02 mol). The two solutions were mixed, kept under stirring at 50 °C for 2 h, and cooled down at room temperature. The organic fraction was recovered by centrifugation at 3000 rpm for 5 min. A final drying was carried out at 40 °C for 48 h, under vacuum (yield 95.0%).

The halide presence was monitored on the organic fraction, according to the Silver Nitrate Test. The complete exchange of the counter-ion was verified by analyzing aliquots of the organic fraction after the silver nitrate addition. The presence of a precipitate indicated that the exchange was not complete. For instance, in the case of the HdmimBr, this condition required that additional water solution, containing the NaDMSIP salt, was reacted with the organic layer to complete the anions exchange. Therefore, no residual halides were present in the produced ILs. Moreover, MALDI TOF analysis was an important tool not only to verify that the metathesis between the bromide/chloride synthetized with the chosen inorganic salts took place, but also to check the presence of halides (unreacted bromide/chloride) in the synthetized ILs. Indeed, no adduct peaks related to the presence of unreacted HdmimBr (expected peak at 695.76 *m/z*) and OOMmimCl (expected peak at 486.15 *m/z*) were found in the MALDI TOF spectra of the synthetized ILs.

On the contrary, the MALDI TOF analysis of ILs with very low yields showed peaks related to the simultaneous presence of the unreacted halides and the IL-synthetized adducts.

#### 2.2.2. Membrane Preparation

The casting solution was prepared by dissolving the Pebax^®^Rnew pellets into ethanol at a concentration of 2 wt%. A clear solution was obtained under reflux conditions for ca. 2 h. Weighed amounts of ILs were added directly to the cooled polymer solution. The ILs were both solid at room temperature.

The membranes were prepared as dense flat sheets by casting the polymer/IL mixture within a stainless-steel ring placed onto a Teflon support. The ring was left at room conditions for a slow solvent evaporation. Isotropic films based on the neat polymer were also prepared and used as reference. The final membranes contained various IL concentrations (1, 5, 10 and 20 wt% of the total membrane weight).

#### 2.2.3. Membrane Characterization

##### Fourier Transform Infrared (FT-IR)

Functional groups of the dense films were investigated by attenuated total reflection FT-IR analysis. The spectra were recorded in a FT-IR Spectrum One (Perkin Elmer, Milan, Italy), in the region of 4000 to 650 cm^−1^, with a resolution of 4 cm^−1^ (16 scans). The same instrument was used in the transmission mode to analyze the neat ILs in the form of solid powders at room conditions by preparing KBr pellets in a hydraulic press.

##### Thermogravimetric Analysis (TGA)

Dynamic TGA curves were obtained under a nitrogen atmosphere at a purge flow of 60 mL/min using thermogravimetric equipment (Q500, TA Instruments, Milan, Italy). Samples (4–5 mg) were heated from 40 to 600 °C, at a heating rate of 10 °C/min.

##### Differential Scanning Calorimetry (DSC)

The melting and crystallization temperatures, as well as the glass transition temperatures, were measured by DSC, under a nitrogen flow, using a Q100 calorimeter (TA Instruments, Milan, Italy); this was supplied with a liquid sub-ambient accessory. The calorimetric measurements were performed in the temperature range of −90 to 200 °C, at a rate of 10 °C/min during both heating and cooling cycles. An empty aluminum pan and high purity standards (indium and cyclohexane) were used as reference and for calibration, respectively.

#### 2.2.4. Gas Permeation Tests

The tested samples had a circular effective area ranging from 11.3 to 2.14 cm^2^; their thickness was determined as the average of multiple point measurements taken by a digital micrometer (IP65, Mitutoyo). A fixed volume/pressure increase instrument (Elektro & Elektronik Service Reuter, Geesthacht, Germany) was used for the gas permeation tests with pure gases (H_2_, He, O_2_, N_2_ and CO_2_) [22]. The feed pressure was 1 bar and the increasing gas pressure at the permeate side was monitored over time in a calibrated volume. The gas permeability (*P*) was evaluated from the slope of the pressure vs. time curve:

(1) $$p\left(t\right)=p\left(t=0\right)+{\frac{dp}{dt}|}_{t=0}t+\frac{RTA}{V_{p}V_{m}}\frac{p_{f}P}{l}\left(t-\frac{l^{2}}{6D}\right)$$

where *p_t_* is the permeate pressure at time *t*, *p_0_* is the starting pressure, (*dp/dt*)_0_ is the baseline slope, *R* is the universal gas constant, *T* is the absolute temperature, *A* is the exposed membrane area, *V_P_* is the permeate volume, *V_m_* is the molar volume of the permeating gas (at standard temperature and pressure, 0 °C and 1 atm), *p_f_* is the feed pressure and *l* is the membrane thickness. The membrane samples were evacuated with a turbo molecular pump to remove previously dissolved species before each test. This step is particularly important also on fresh membrane samples, which can contain traces of residual solvent or gases adsorbed form the ambient. Indeed, ILs tend to pick up moisture from the environment and the water content of ILs has a crucial influence on their properties. Defect-free samples, that are well evacuated, have a negligible $p\left(t=0\right)+{\frac{dp}{dt}|}_{t=0}$ term.

The gas time lag (*θ*), obtained by extrapolating the transient linear portion of the pressure curve on the abscissa, is used to estimate the diffusion coefficient (*D*) of each gas through the membrane [23]:

(2) $$D=\frac{l^{2}}{6\theta }$$

The ‘solution–diffusion’ model that describes the transport in dense polymeric films [24] was considered to calculate the solubility coefficient (*S*) as follows:

*S* = *P*/*D* (3)

The ideal selectivity was obtained as the ratio of the specific permeability values for two gases. It can be decoupled into solubility selectivity and diffusion selectivity:

*α*_A/B_ = *P*_A_/*P*_B_ = *S*_A_/*S*_B_ × *D*_A_/*D*_B_ (4)

## 3. Results

Both ILs were obtained as powdered materials at room temperature.

Homogeneous defect-free films were obtained with an average thickness of ca. 100 micron.

**Figure 2:**
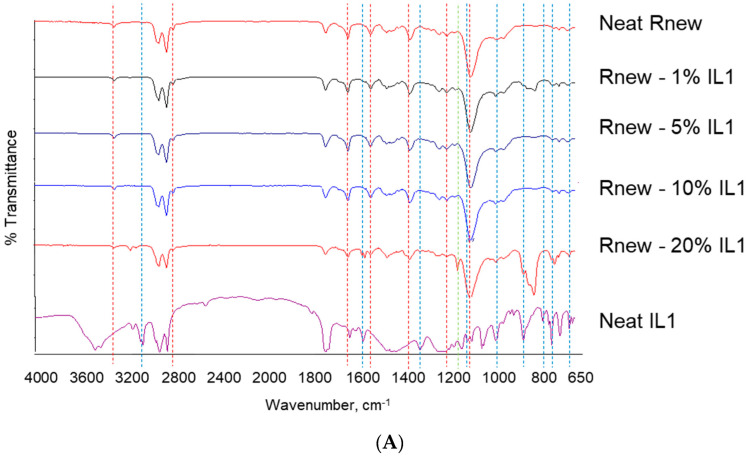

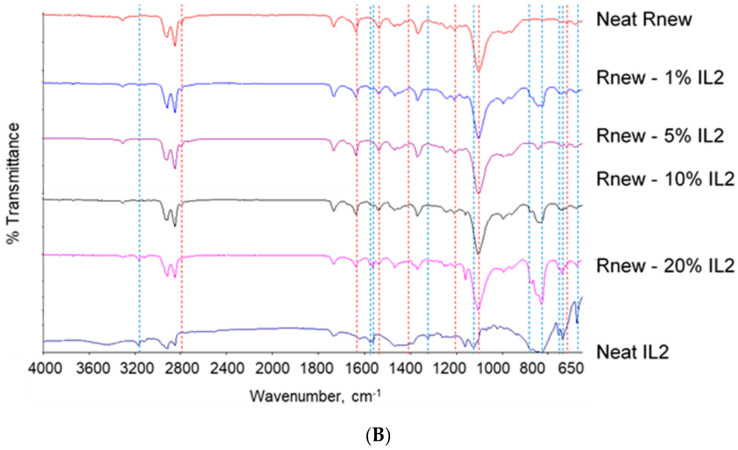
FT-IR spectra for Pebax^®^Rnew and Pebax^®^Rnew composite membranes. (**A**) Samples containing IL1 (HdmimDMSIP); (**B**) Samples containing IL2 (OOMmimPF_6_). Red dashed lines identify characteristic bands of the neat copolymer, while blue dashed lines identify characteristic bands of the neat ILs.

The synthetized ILs and the as-prepared film samples were analyzed by FT-IR and the obtained spectra are shown in Figure 2. The neat Pebax film displays strong bands at around 1100 and 1640 cm^–1^; these are related to the C–O–C (ether group) stretching vibrations within the PTMO block and to the H–N–C=O stretching vibrations in the polyamide, respectively. Another characteristic band for the pure Pebax^®^ membrane is that which is assigned to the stretching vibrations of C=O at 1735 cm^–1^ [25], while the peak at 3300 cm^–1^ derives from the N–H stretching vibrations in the polyamide. The peaks at 2921 cm^−1^ and 2852 cm^−1^, related to the aliphatic symmetric and asymmetric (C–H) stretching vibrations caused by the methyl groups in the polymer [25], are also characteristic of the selected ILs [26].

Both ILs present a –C=N stretching vibration band at around 1635 cm^−1^ that is also distinctive of the polyamide block in the Pebax matrix.

The sulfone group in the anion of IL1 presents bands at 1235 cm^−1^ (asymmetric vibration of S=O) and around 1060 cm^−1^ (symmetric vibration of S=O), as shown in Figure 2A. The peak of 830 cm^−1^ is a characteristic frequency of PF_6_^−^, while the band at ca. 1130 cm^−1^ indicates the C-O-C ether stretching in the Imidazolium side chain (Figure 2B).

The composite samples, particularly in the fingerprint region, display characteristic peaks that are due to the Imidazole ring of the ILs. The band at 1161 cm^−1^ is related to the Imidazole H–C–C and H–C–N bending and the band at 752 cm^–1^ is characteristic of the out-of-plane C–H bending of Imidazole rings [27].

Physical interactions are evident in the nanocomposite films. The C atom between the highly electronegative neighboring two N atoms in the Imidazole ring of the IL cation can form hydrogen bonds [28]. The band characteristic of the in-plane Imidazole ring bending, which typically has a peak at 840 cm^−1^ [27], is split into two bands (at 828 cm^–1^ and 855 cm^–1^) in the IL1 nanocomposites. They can be considered as hydrogen-bonded and free state (at higher wavelength), respectively, with a more intense bonded part. The same behavior is observable in both types of nanocomposites for the band at ca. 1570 cm^–1^ (Imidazolium ring stretching [27]), which is split with intensity variations (at 1564 and 1576 cm^–1^). Such splits can be attributed to specific interactions of the Imidazolium cation with the polymer matrix. In addition, the band for the out-of-plane C–H bending of the Imidazole ring has another peak at 739 cm^−1^ due to the hydrogen-bonded state.

The polar PA block can form hydrogen bonds with smaller polar molecules [16]. The double peak, observed in the polymer matrix at 1469 cm^–1^ and assigned to the C=O stretching vibration in carbonyl, has a more intense hydrogen-bonded part in the blends, indicating hydrogen bonding with the polyamide segments in Pebax. The peak at 1538 cm^−1^ (bending of N–H in the PA block in Pebax [29]) is slightly blue-shifted at 1541 cm^–1^ in both blends, revealing a bond contraction in the composite membranes. This indicates the high energy of these bonds in the polymer matrix.

As previously reported [20,21], the TGA thermograms, obtained during the dynamic degradation tests for both ILs, display a single degradation step, reaching the maximum temperature for decomposition at 411 °C (IL1) and at 271 °C (IL2). The decomposition onset temperature (*T*onset), a worthy descriptor of the thermal stability of a material, was 340 °C for IL1, whereas it was 240 °C for IL2, indicating a higher stability of IL1 than IL2; this is probably due to both the hygroscopicity of IL2 and the presence of an alkoxylated side chain in its Imidazolium cation [21].

**Figure 3:**
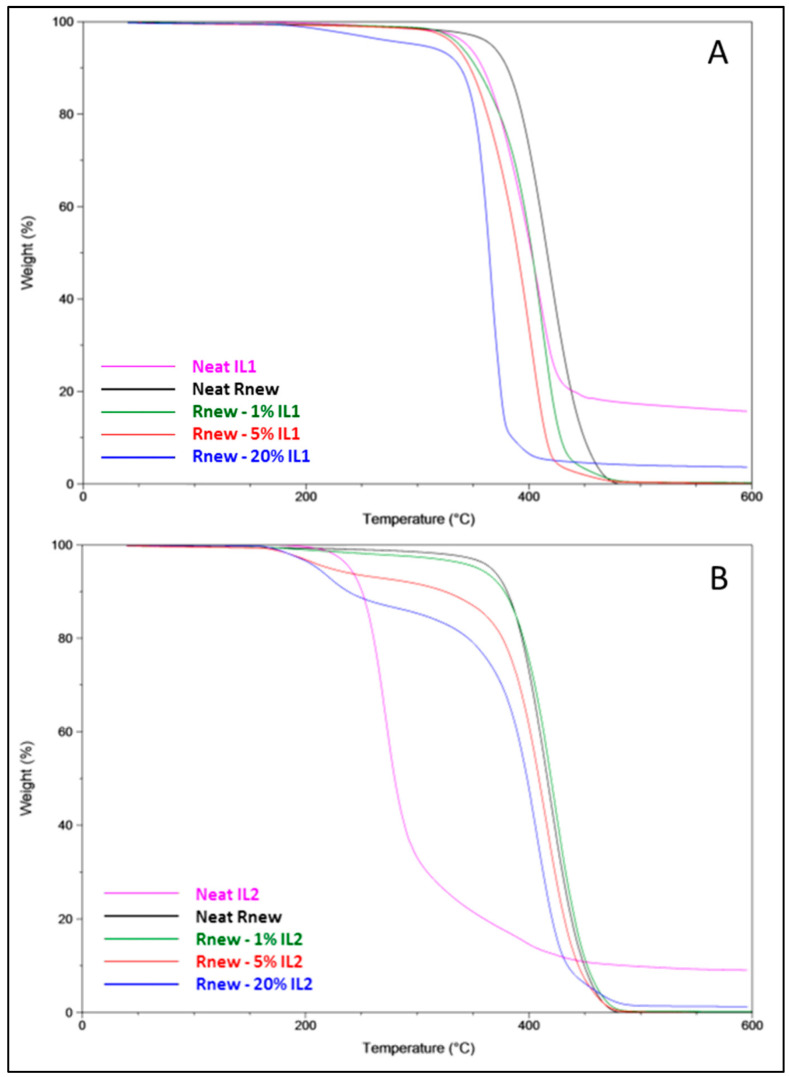
TGA thermograms for Pebax^®^Rnew and Pebax^®^Rnew composite membranes. (**A**) Samples containing IL1 (HdmimDMSIP); (**B**) Samples containing IL2 (OOMmimPF_6_).

**Figure 4:**
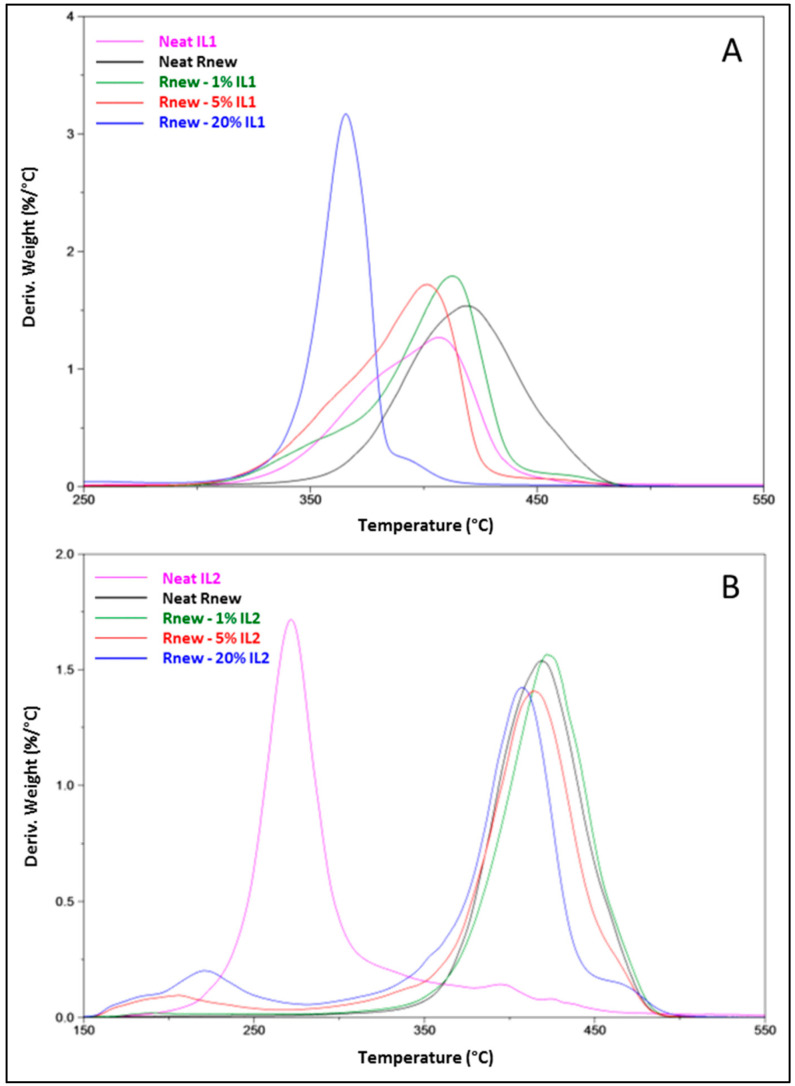
DTGA thermograms for Pebax^®^Rnew and Pebax^®^Rnew composite membranes. (**A**) Samples containing IL1 (HdmimDMSIP); (**B**) Samples containing IL2 (OOMmimPF_6_).

The pristine Pebax®Rnew film and almost all its blends displayed a single degradation step, with no significant differences below 200 °C, indicating that the loading of the ILs does not affect the thermal stability of the polymer (Figure 3). Nevertheless, at temperatures above 200 °C, a decreasing trend in degradation temperatures was observed. In particular, the pristine polymer showed a *T*onset at ca. 366 °C that shifted at lower temperatures according to the IL concentration in the blend films. Indeed, reductions of 29–66 °C and 12–155 °C were observed for the onset temperature in the films containing IL1 and IL2, respectively. The drastic decrease shown by the films containing 5–20% IL2 is probably due to absorbed water and residual solvent trapped in the polymer matrix, even after rigorous drying in a vacuum oven. This was confirmed by the peaks in the range 210–230 °C, evidenced in the DTG curves (Figure 4B).

The maximum degradation temperature (*T*_d_), as well as the temperature at a 50% weight loss in the blend films, showed a less pronounced decreasing behavior than the onset temperature, displaying reductions up to ca. 50 and 10 °C for the films containing 20% IL1 and IL2, respectively (Table 1).

The DTG curves highlight the shift for the peak corresponding to the polymeric matrix at lower temperatures (Figure 4). Typically, the decomposition temperature of the IL decreases for longer alkyl chains on the cation [30]. In our study, the Rnew–IL1 films showed a clear shift to lower temperatures, particularly evident for the 20% concentration, whereas minor shifts were observed in the Rnew–IL2 blends. A shoulder, observed at ca. 390 °C (Rnew–20%IL1) and at 430 °C (Rnew–20%IL2), is indicative of a complexation between the polymer and the ILs [31]. This shoulder was more outlined in the sample containing 20% IL2. Moreover, the peaks detected at 210–230 °C (Rnew–5–20% IL2) are due to the hygroscopic behavior of IL2 and the solvent residue from film preparation, as mentioned above.

The residue of the polymer films is not different with respect to that of neat Pebax, except for the films containing an IL concentration of 20%. In these blends, a slight increase in the residue is observed, probably due to the complexes between the ILs and the polymer, which do not completely degrade up to 600 °C.

The temperature ramp rate of 10 °C/min, used in the dynamic TGA under nitrogen, was the same as that adopted in the DSC analyses.

**Figure 5:**
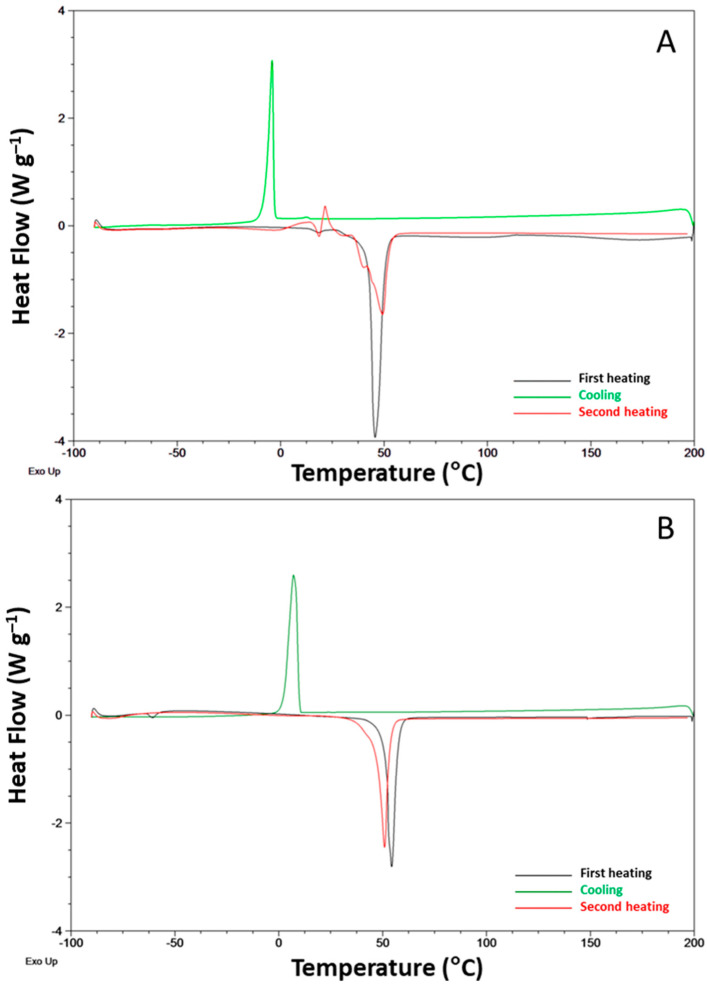
DSC traces for the neat ILs. (**A**) IL1 (HdmimDMSIP); (**B**) IL2 (OOMmimPF_6_).

The DSC investigation on the neat ILs evidenced distinct melting points upon heating at ca. 49 °C and 52 °C for IL1 and IL2, respectively. Their freezing point was considerably lower, at −4 °C for IL1 and 7 °C for IL2 (Figure 5). No glass transition temperatures (*T*g) and no significant differences in IL melting (*T*m) and crystallization (*T*c) were found when comparing the first and second heating runs. However, IL1 displays a multifaceted phase behavior (Figure 5A) compared to IL2 (Figure 5B), as previously reported [20,21]. Indeed, in the second heating, a polymorphic behavior, characterized by cold crystallization and endothermic transitions, likely due to the melting of different crystals or solid-solid transitions, was found [20,21].

**Figure 6:**
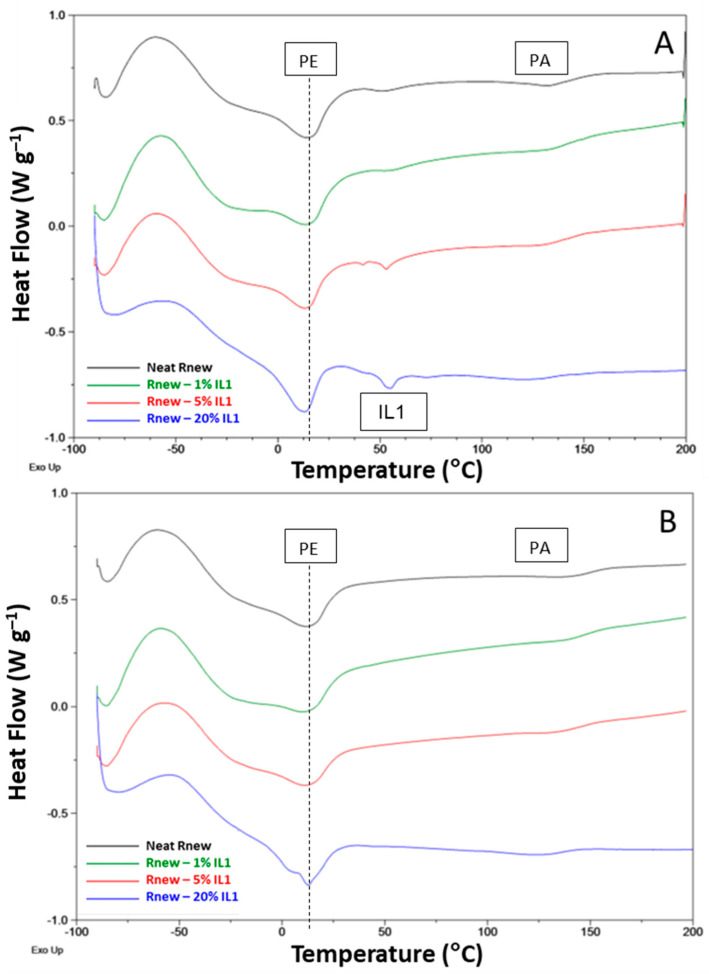
DSC traces for neat Pebax^®^Rnew and Pebax^®^Rnew–IL1 (HdmimDMSIP) composite membranes. (**A**) First heating scan; (**B**) Second heating scan.

**Figure 7:**
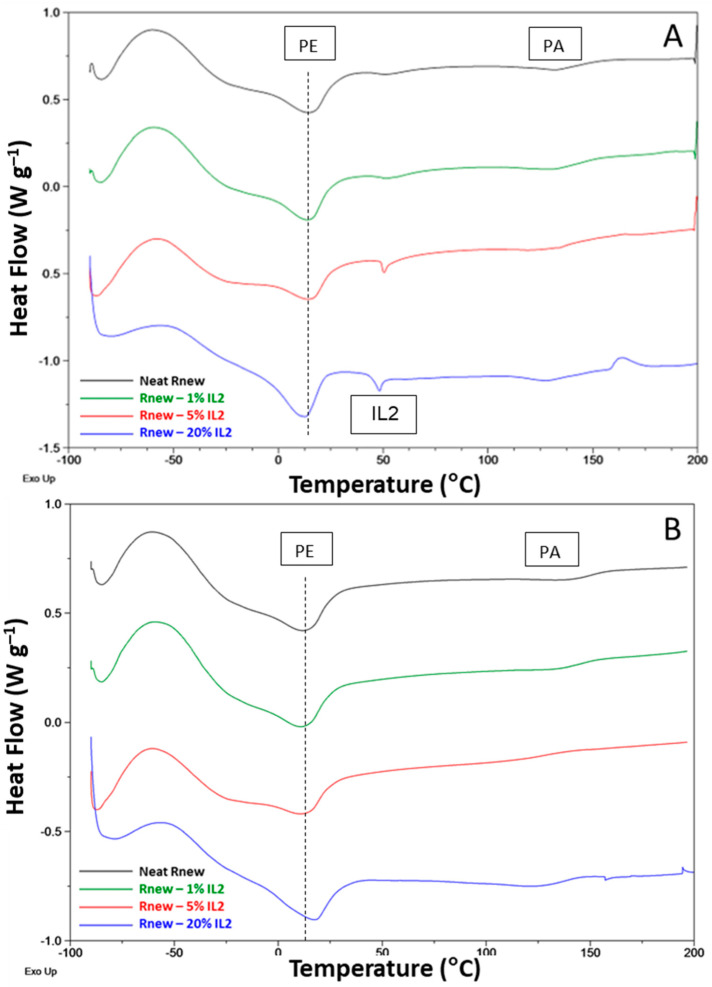
DSC traces for neat Pebax^®^Rnew and Pebax^®^Rnew–IL2 (OOMmimPF_6_) composite membranes. (**A**) First heating scan; (**B**) Second heating scan.

As shown in Figure 6 and Figure 7, the first heating scan for the neat Pebax membrane presents the melting peaks for the PA11 block and PTMO at ca. 140 °C and 13 °C, respectively. Indeed, the hard PA11 segment is reticulated by hydrogen bonding, resulting in a rigid crystalline block with a high melting temperature. The Pebax soft segment has a low *T*g, of ca. −39 °C. This scan displays the behavior of the films prepared from the solution casting, which were not subjected to thermal cycles.

In all samples, at ca. 60 °C, a broad peak was observed, probably due to a kinetically less favorable crystal phase induced by the slow solvent evaporation during membrane preparation. This peak and the melting peaks of both ILs loaded at high concentrations into the polymer matrix disappeared during the second heating run, suggesting that these crystalline phases form in the films from the solution state containing solid ILs, while a different behavior is found for the blends analyzed in the molten state. A slight shift to high temperatures (16 °C) for the *T*m of the PTMO block and 144 °C for the PA block was observed.

Despite the different composition, the selected ILs display similar temperatures in their thermal transitions (see DSC analysis, melting temperature at ca. 50 °C), enabling their characterization in the same temperature range to study the effect of this solid–liquid transition on the resulting gas permeation properties of the prepared films. The heat capacity (*c*p) of the two ILs was calculated on the basis of two correlations available in the open literature [32,33]. According to Ahmadi et al. [32], the *c*p values calculated at 25 °C are 1114 and 587 J/(mol K) for IL1 and IL2, respectively. Similar values are obtained applying the correlation developed by Farahani et al. [33]: 1097 and 562 J/(mol K) for IL1 and IL2, respectively. These calculations well match the experimental data available for Imidazolium-based ILs, since by increasing the side alkyl chain in the cation and, in general, the molecular weight of the salt, the thermal capacity and also the viscosity increase.

Both ILs induce a reduced crystallinity for the predominant PTMO phase, as testified by the decrease in the associated melting enthalpy (Δ*H*m), particularly in the films containing a higher concentration of ILs. Moreover, the PTMO has a melting point below room temperature; consequently, it is amorphous at higher temperatures.

**Figure 8:**
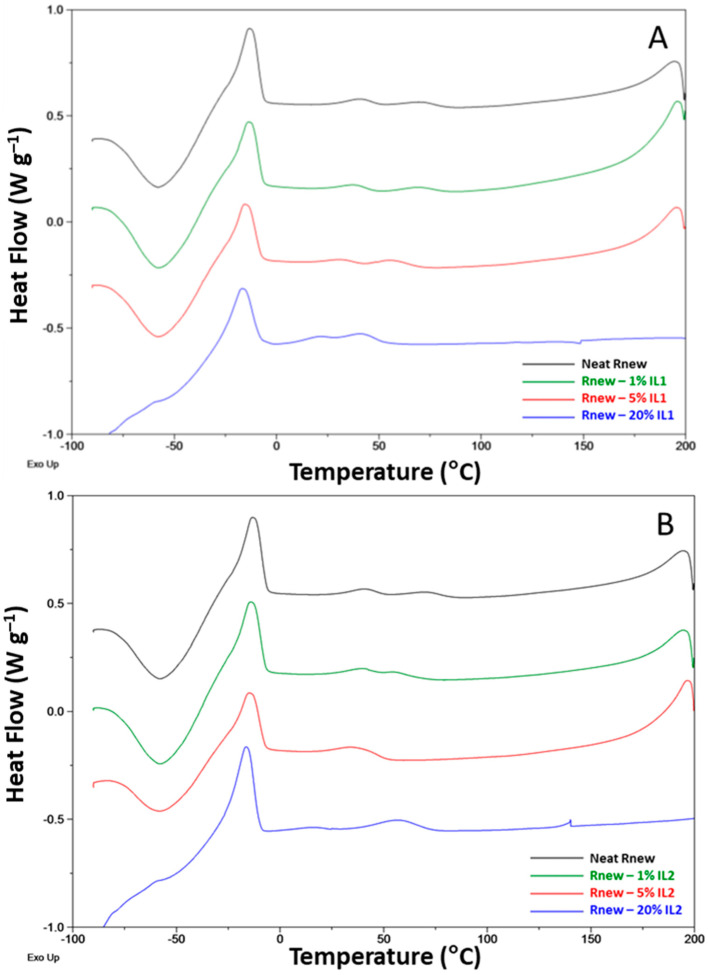
DSC traces for neat Pebax^®^Rnew and Pebax^®^Rnew composite membranes. Cooling scan. (**A**) Samples containing IL1 (HdmimDMSIP); (**B**) Samples containing IL2 (OOMmimPF_6_).

Crystallization peaks are clearly visible at a reduced temperature in the cooling cycle (Figure 8) for the Pebax blocks (ca. 40 °C PA11 and −14 °C PTMO). These peaks shift to slightly higher temperatures, with a 2-4 °C increase for both blocks in the blend films.

In general, IL addition to Rnew did not influence the positions of the melting and crystallization peaks; the slight shifts (1–5 °C) detected suggest no interaction and, consequently, phase separation between the polymer and the ILs.

The characteristic peaks of the copolymer relative to the melting for the polyamide and polyether blocks are preserved in the composite films; however, the *T*melting is only slightly influenced upon the IL addition.

In the blends, the *T*g values of the PTMO segment showed an increase of 1–3 °C upon the loading of the ILs with respect to the pristine polymer, indicating a reduced mobility in the amorphous polymer block, particularly in the IL1 samples at a high concentration. As a consequence, the polymer’s stiffness and chain mobility are affected. Indeed, *T*g is a cohesive energy parameter [34]. Moreover, the ILs in the composite membranes have a phase transition (*T*melting) of 55 °C for IL1 and 48.1 °C for IL2 (first heating scan). The previous thermal history was removed by heating them to above their melting point. Considering the cooling scan, no crystallization peak related to the ILs was present in the nanocomposite films. Thus, no supercooling was evident for both ILs once incorporated in the membrane. Accordingly, no melting peaks can be observed for the IL phase in the second heating trace for both ILs. In the case of the Rnew–IL1 blend at 20%, the PTMO melting is anticipated, as evidenced by a shoulder on the melting peak at around 4 °C due to metastable crystals that melt before the more stable ones.

Therefore, the film formed via solvent casting, represented by the first heating trace, is different from that obtained from the molten state after the polymer heats up to a high temperature (ca. 200 °C) and cools at the rate of 10 °C/min.

The gas permeation tests provided the main transport parameters: permeability, diffusion, solubility and the ideal selectivity for each of them. The data measured at 25 °C on the as-prepared samples are reported in Table 2. The considered permanent gases, with a molecular size below 1 nm, act as molecular probes for the membrane microstructure. At a certain temperature, the gas permeability is indicative of the membrane morphology, the polymer structure (e.g., degree of crystallinity) and chemistry (e.g., ratio of the two blocks), as well as the polymer/permeant interactions.

The gas permeation data confirm the dense nature of all the prepared films. Due to the large amount of the flexible PTMO segments in the copolymer, Pebax®Rnew presents a sustained gas permeability [20]. Table 3 proposes a comparison with other Pebax^®^ grades, showing a reduced gas permeability when the amount of the PE block is lowered. Indeed, the melting point for the crystalline part of the polyether block is below room temperature, as indicated by DSC. In the neat Pebax^®^, the predominant polyether block is responsible for a preferential affinity for CO_2_, compared to nonpolar gases such as N_2_ and H_2_ [35].

In the case of the composite membranes, the amount and type of the IL also affects the permeation parameters and the transport mechanisms (Table 2). At room temperature, the addition of increasing quantities of both ILs to Pebax^®^ progressively decreased the overall gas permeability. Therefore, the solid ILs acted as impermeable fillers that were incorporated in the polymer matrix. The permeation rates of the tested gases in the composite films follow the same order as in the neat polymer, with CO_2_ being the most permeable species, as in the pristine polymer matrix (Table 2). On the other hand, CO_2_ molecules can also favorably interact with the cationic Imidazolium π system in the added ILs [38]. The nature of the anion in Imidazolium-based ILs has a key role in determining the affinity with CO2 [39,40]. Typically, ILs and PILs that comprise the PF6 anion display a good CO2 solubility [39]. In addition, the Sulfonic group in the DMSIP anion IL1 displays a strong affinity with CO2 via specific dipole–dipole interactions [28,41].

### 3.1. Different Behaviour of the Two ILs

The decline in permeability, observed in the nanocomposites that encapsulate impermeable fillers, originates from more tortuous paths imposed on the permeating gas molecules and/or from filler/polymer interactions [42]. The ratio of the composite membrane permeability to that of the neat matrix (*P/P*_0_) decreases more in the membranes comprising 10% IL2. The presence of the oxygen ether atom in the alkyl side chain of the IL2 cation could be responsible for stronger interactions with the polymer, as inferred by the thermal analysis. Moreover, the smaller molecular weight of IL2 implies a larger number of moles and, thus, more ions are present in the IL2-loaded membrane samples at the same weight loading of ILs. In addition, its anion (PF_6_) is poorly nucleophilic and classified as a non-coordinating anion, resulting in a diminished anion–cation interaction that enables interactions with the polymer matrix. On the other hand, PEO polymers are capable of strongly interacting with ILs, promoting a cation–anion dissociation in the IL salt via O^−^–cation interactions [43]. Similar interactions can be expected for the prepared nanocomposite membranes in which the PTMO block is predominant, as indicated by the PTMO *T*g that is shifted at higher temperatures (Figure 6).

Typically, ILs bearing a longer alkyl side chain on the cation are characterized by a larger free volume amount [44]. This feature was detected in Imidazolium ILs with increasingly bulkier anions by Xenon NMR [45] and by Molecular Dynamics studies [46]. Molecular simulations showed larger voids in these ILs due to the reduced ion attraction [47]. A weak interionic interaction enables larger distances between ions, facilitating the insertion of CO_2_ between these ions [47]. The bulkier anion in IL1 favors the ion displacement. Therefore, larger cages are expected in the neat IL1, counteracting the permeability depression due to the more rigid polymer matrix. However, at a high IL concentration (e.g., 20 wt%), the ILs hardly maintain the supramolecular assembly that characterizes the neat materials, and important permeability reductions can be observed.

**Figure 9:**
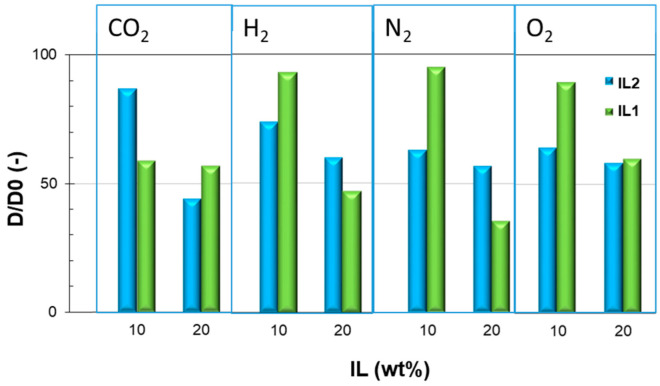
Gas diffusion coefficient in as-prepared Pebax^®^Rnew composite membranes relative to neat Pebax^®^ (*D/D*_0_). *T* = 25 °C.

The gas diffusion coefficient also evidences a clear decreasing trend upon the addition of ILs to Pebax compared to the pristine polymer matrix (Figure 9). The augmented cohesive energy density for the PTMO block in the nanocomposite films, indicated by its higher *T*g, hinders the gas diffusion.

### 3.2. Temperature Effect

The gas permeability through the Pebax membranes, as well as in all Pebax/ILs films, increased with temperature. However, gas sorption in polymers depends sensitively on temperature, and the predominant solubility contribution for CO_2_ is depressed by increasing the temperature. Accordingly, a higher CO_2_/N_2_ selectivity can be achieved in the Pebax matrix by reducing the operating temperature. Thus, the gas transport mechanism is diffusion-dominated in the temperature range tested.

**Figure 10:**
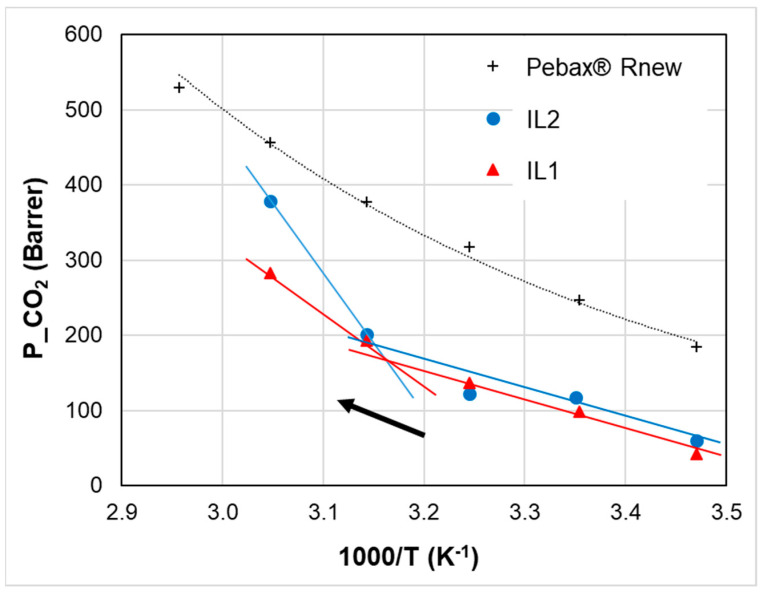
Temperature effect on CO_2_ permeability for Pebax^®^Rnew and Pebax^®^Rnew–IL composite membranes at 20% of IL loading. Heating mode. Lines are a guide for the eyes.

The temperature-dependent permeation in the nanocomposite membranes differs from the neat copolymer, as shown in Figure 10, in the case of CO_2_ for the blends loaded with 20% of ILs. Gas transport properties show a discontinuity at a temperature close to the melting point of the ILs (i.e., 50 °C as observed in the DSC trace). Below their melting point, the solid long chain ILs reduce the permeability of the polymer. Above this temperature (1000/*T* ≈ 3.1 K^−1^), there is a jump in the gas permeability coefficient. A similar discontinuity was reported for the ionic conductivity of a piperidinium IL and attributed to the IL solid–liquid transition upon heating [43].

Moreover, by changing the IL, different temperature-responsive behaviors can be established for the same copolymer (Figure 10). In particular, above the IL melting, IL2 is more efficient in increasing the gas permeability at higher temperatures.

An increased temperature substantially diminishes the intensity of intermolecular interactions, reducing the IL viscosity. Indeed, the viscosity is significantly enhanced in ILs with a large cation size. This is particularly evident in asymmetric cations [C*_N_*_–1_C_1_im] above N = 6, due to the increase in the conformational entropy and the possibility of alkyl chain folding [48]. Thus, the considered ILs are highly viscous in the liquid state, reflecting the high molecular weight, as well as the intermolecular interactions (e.g., H–bonding, dispersive and electrostatic interactions), particularly IL1.

The temperature dependency of permeation parameters in the neat Pebax membrane can be approximated by an Arrhenius expression:

*P* = *P*^0^ exp(−*E*p/*RT*) (5)

where *P*^0^ is a pre-exponential factor and *E*p is the apparent activation energy for permeation [49].

An Arrhenius behavior is observed above and below the IL melting point in the nanocomposite films. Thus, two activation energies were evaluated (Table 4). As stated for thermodynamic properties of ILs, in order to have reproducible data, they should be collected in heating modes [50]. Therefore, we took the data from heating scans to obtain the activation energies of the different samples.

The *E*p values in the nanocomposite membranes are higher than in the neat Pebax film (Table 3), indicating their more pronounced sensitivity with respect to temperature changes, but also a denser structure.

In all prepared films, the order of *E*p was: N_2_ > O_2_ > H_2_ ≈ He > CO_2_ (Table 3), showing the greatest influence of temperature on the less permeable species (e.g., N_2_). Accordingly, the permselectivity for large/small gas pairs is generally depressed by increasing the temperature because the less permeable species present a higher activation energy than more permeable molecules.

At a concentration of 10%, the smaller IL2 induces significant changes in the *E*p in the whole range of investigated temperatures, whereas the bulkier IL1 does not evidence significant differences with respect to the neat polymer. This is consistent with the polymer/IL interactions that are expected to be more significant in the molten state for IL2, which brings the ether oxygen on the cation side chain, as also evidenced by the DSC and TGA analysis. At a loading of 20%, *E*p2 remains still higher for IL2 for all the tested gas species, while *E*p1 becomes higher for films incorporating IL1.

### 3.3. Thermal Cycling

To examine the thermal response of the fabricated membranes, the operating temperature was changed in a cyclic way during the permeation tests, considering ranges that include the IL liquid–solid transition temperature (*Tm*). A different temperature sensitivity in the nanocomposites was demonstrated with respect to pristine Pebax membranes.

**Figure 11:**
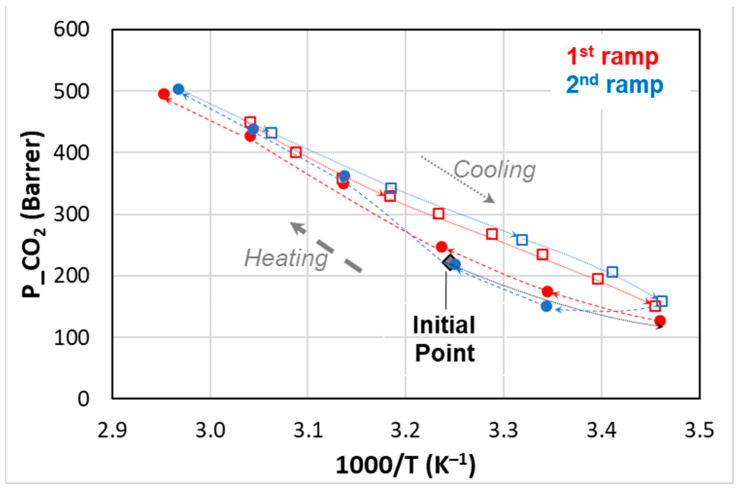
Temperature effect on CO_2_ permeability for Rnew–10% IL2 composite membrane. Closed symbols: heating; open symbols: cooling.

A permeability hysteresis was established for CO_2_, as shown by also recording the permeation fluxes during the cooling in a temperature range that included the IL *T*m. Figure 11 describes the behavior of the sample loaded with 10% IL2 upon heating and cooling that was carried out with comparable heating and cooling rates.

Starting from the solid state of the IL (*T* = 35 °C), the membrane was first cooled at 15 °C and then its permeability was measured by increasing the temperature. No permeability hysteresis was found when the changes were below the IL *T*m.

Upon heating, the CO_2_ permeability increases forming a knee: a low slope below the IL *T*m and a steeper behavior above IL *T*m. Instead, upon cooling from a temperature > *T*m, the permeability decay was quite linear in the plot *P* vs. *1/T*, and the experimental points were located above those measured in the heating ramp. Thus, the film was more permeable in the *T* range below the melting of the IL when the low *T* was reached from the melt state of the IL.

The data are reproducible when repeated heating and cooling ramps are carried out.

**Figure 12:**
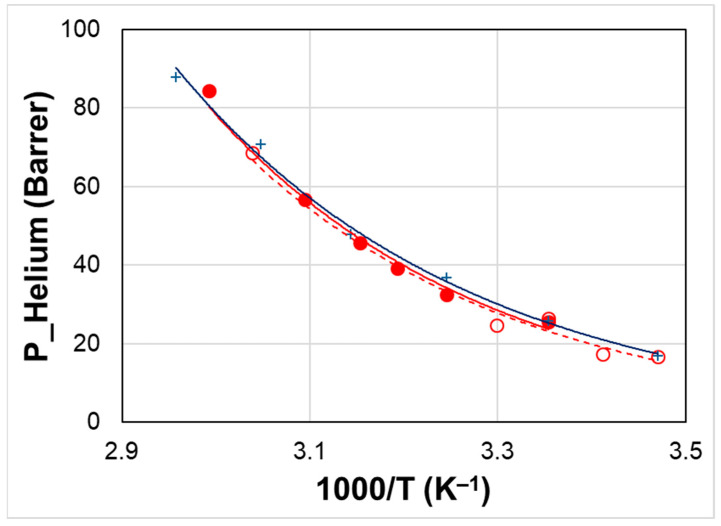
Temperature effect on Helium permeability for Rnew (plus sign, +) and Rnew–10% IL1 (HdmimDMSIP) composite membranes (circle). Closed symbols: heating; open symbols: cooling.

However, the membrane microstructure reached upon cooling allows the permeation of small gases that experience a lower resistance than larger molecules. Indeed, no hysteresis is observed as far as small gas molecules are considered (e.g., He and H_2_) since the permeability curves in the first and second heating ramp are quite similar (Figure 12). Thus, the selectivity of the membrane can be changed depending on the heating or cooling.

**Figure 13:**
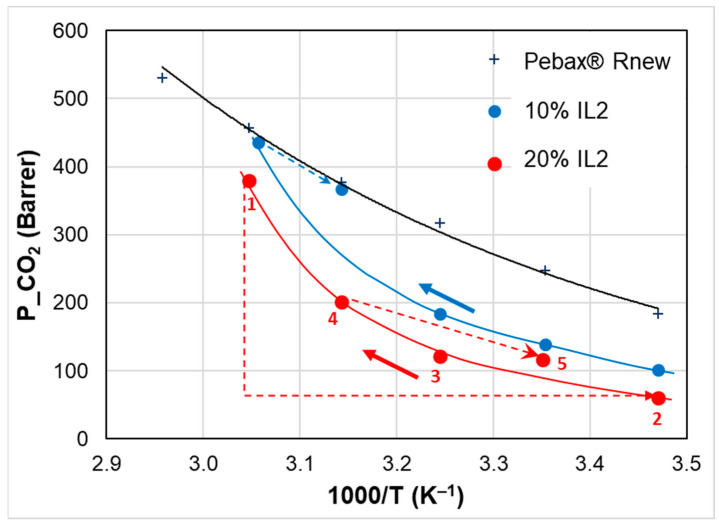
Temperature effect on CO_2_ permeability for Rnew (plus sign, +) and Rnew–IL2 (OOMmimPF_6_) composite membranes at 20% (red circles) and 10% IL (blue circles).

Other tests carried out with CO_2_ showed more significant hysteresis, as evidenced for the sample containing 10% IL2 and attaining permeability values very close to those of the neat polymer upon rapid cooling (Figure 13). A comparison is presented with the film containing 20% IL2 performing the cooling below the IL *T*m, from 45 to 25 °C.

These tests (Figure 11 and Figure 13) show that the rate of the temperature change affects the material response. Indeed, ILs can display complex thermal behavior owing to their nanostructuration, which is determined by charge neutrality constraints and the segregation of their non-polar long alkyl chains [50]. Compared from atomic and molecular liquids, ILs present an internal degree of freedom, permitting conformational changes with *T* [51]. However, solidification kinetics are commonly slow for ILs. Accordingly, a high rate in the *T* variation prevents the gel membrane from returning to its initial structure, remaining quite permeable. On the contrary, a different behavior can be established by keeping the composite samples at low *T* for long equilibration times, as evidenced by the low permeability values (Figure 12). Indeed, upon cooling from the liquid state, the ILs may form a glass at a low temperature [50].

**Figure 14:**
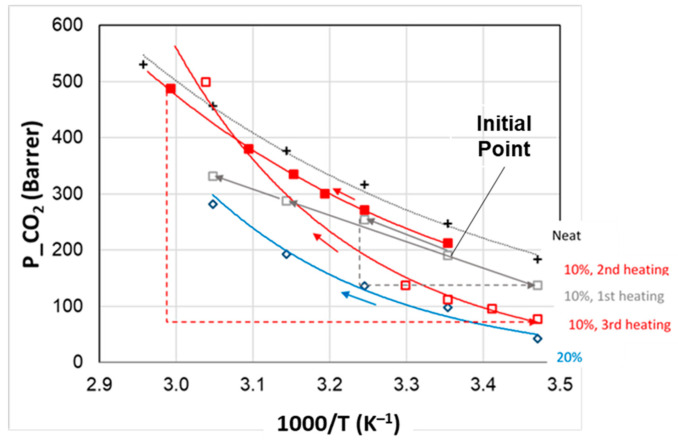
CO_2_ permeability measured during heating ramps in Rnew–10% IL1 composite membrane compared to neat Rnew and Rnew–20% IL2 composite membrane. Lines are the exponential fit of the experimental points.

Figure 14 shows the response to repeated temperature cycles of the membrane containing 10% IL1, recording the data during the heating ramps, performed at different rates.

The CO_2_ permeability measured on the as-prepared membrane in the first heating cycle (from 25 to 35 °C, then cooling at 15 °C and heating up to 45 and 55 °C) was quite below the value of the neat polymer, reflecting a more impermeable structure.

In a second heating cycle (from 25 to 60 °C), the permeability values at 25 and 35 °C were confirmed, while at a higher temperature, the permeation fluxes were larger and closer to those measured for the neat Pebax. Indeed, this ramp was carried out by taking more measurements and, thus, the changes are gradual and allow an equilibration of the sample at each temperature after small variations. After cooling from 60 to 15 °C and keeping the temperature low overnight, during the third heating, the sample presented a quite low permeability in the range 15–30 °C, close to the data obtained for the membrane containing 20% IL1. In this case, the microstructure of the sample was rearranged in a different way with respect to the as-prepared sample. Indeed, upon a slow cooling from 65 to 15 °C, with a prolonged time at low temperature, a denser structure with a redistribution of gaps was obtained. Instead, the membranes were obtained. As also evidenced by DSC traces recorded during heating and cooling, the as-prepared film, formed after the slow solvent evaporation of a solution that was magnetically stirred, reaching a certain dispersion of the IL, and used for the first heating cycle, behaved differently compared to the same film upon cooling from a melt IL state.

However, the high permeability can be recovered by the slow heating of the composite sample from 30 to 55 °C. Indeed, once above the IL *T*m, the gas permeability evaluated at 55 °C surpassed the neat polymer line. This demonstrates how the rate of the thermal changes has a relevant role in the temperature-responsive behavior of the developed composites that are based on the combination of a rubbery polymer and ILs with a low solid–liquid temperature.

## 4. Conclusions

Temperature-responsive composite membranes were prepared via the solution casting technique, incorporating two synthetized ILs (IL1, HdmimDMSIP and IL2, OOMmimPF_6_) within a rubbery polymer matrix of Pebax^®^Rnew, based on a bio-polyamide. The ILs present the Imidazolium group with long alkyl side chains on the cation and are solid at room temperature (solid–liquid transition around 50 °C). Both the polymer matrix and the selected ILs are interesting for separating polar and non-polar gas pairs.

The salts have a barrier effect on the gas permeation and a permeability decline is observed for the as-prepared nanocomposites. The performance of the nanocomposite films can be modulated by changing the IL type and its amount. A thermal analysis of the nanocomposite membranes indicates a somewhat reduced thermal stability upon the loading of the ILs and a complexation of IL2, having an oxygen in the alkyl side chain of the cation with the polymer matrix. The two blocks are differently affected by the ILs. FT-IR proves that the added ILs interact with the PA block in the copolymer matrix. The melting temperature for the crystalline part of the PA block is reduced upon the IL loading, while the glass transition temperature for the predominant soft PTMO block increases.

Gas permeation evidences a temperature-responsive behavior in the films loaded with both ILs, with a discontinuity as the temperature approached the IL structural transition. The temperature dependence for the gas permeability follows an Arrhenius-type thermally-activated behavior with two activation energies, below and above the melting of the IL.

Differently from small molecules such as He and H_2_, permeability hysteresis can be found for large and soluble molecules such as CO_2_, due to temperature-responsive changes in the membrane microscopic morphology that originate from the hierarchical structure of the ILs and the presence of long alkyl chains in the ILs. However, no defects are evident at a low temperature, at which the ILs are in a solid state upon the repeated thermal cycles. Interestingly, the temperature response of the gas transport through the prepared polymer gel/ILs composite membranes can be tailored by simply changing the rate of the temperature variations across the IL solid–liquid transition.

## Figures and Tables

**Table 1:** Thermogravimetric data of neat Pebax^®^Rnew and its ILs blends.

Sample	T_Δm = 5%_ (°C) ^a^	T_Δm = 50%_ (°C) ^b^	T_d1_ (°C) ^c^	% R ^d^
Neat Rnew	366	416	418	0.0
Rnew–1% IL1	337	403	413	0.2
Rnew–5% IL1	333	389	402	0.1
Rnew–20% IL1	300	364	366	3.6
Rnew–1% IL2	354	420	422	0.2
Rnew–5% IL2	222	409	415	0.0
Rnew–20% IL2	211	398	408	1.3

^a^: Onset of degradation (temperature of 5% weight loss); ^b^: Onset of degradation (temperature of 50% weight loss); ^c^: Decomposition maximum temperature of thermal degradation; ^d^: Weight residue (%) at 600 °C.

**Table 2:** Gas permeability measured on the as-prepared Pebax^®^Rnew and Pebax^®^Rnew/IL composite membranes at 25 °C.

Sample	Permeability (Barrer)					Selectivity (–)		
	CO_2_	O_2_	N_2_	He	H_2_	CO_2_/N_2_	H_2_/N_2_	O_2_/N_2_
Neat Rnew	247	23.6	8.9	25.7	45.4	27.9	5.12	2.66
Rnew–1% IL2	232	22.4	8.6	25.7	-	27.0	-	2.60
Rnew–5% IL2	226	21.6	8.1	23.8	38.1	28.0	4.73	2.61
Rnew–10% IL2	139	13.3	5.2	18.4	25.9	27.0	5.01	2.57
Rnew–20% IL2	117	11.5	4.1	17.4	25.1	28.3	6.14	2.77
Rnew–1% IL1	217	21.4	8.5	23.7	-	25.6	-	2.53
Rnew–5% IL1	195	19.2	7.2	24.1	40.3	27.2	5.63	2.68
Rnew–10% IL1	191	18.4	6.86	23.4	36.2	27.8	5.28	2.68
Rnew–20% IL1	98.4	10.4	4.4	13.4	23.2	22.3	5.25	2.35

1 Barrer = 10^−10^ cm^3^ (STP) cm cm^−2^ cmHg^−1^ s^−1.^

**Table 3:** Composite different Pebax^®^ grades and gas permeability for measured in the corresponding membranes.

Grade	Composition	PE	PA	Permeability (Barrer)		Ref.
		wt%	wt%	CO_2_	*T*	
Neat Rnew	PTMO/PA11	79–83	17–21	247	25 °C	[This work]
2533	PTMO/PA12	80	20	276 ^a^ 241 ^b^	30 °C	[36]
3533	PTMO/PA12	70	30	204 ^a^ 256 ^b^	30 °C	[36]
1657	PEO/PA12	60	40	66.5	25 °C	[11]
4011	PEO/PA6	57	43	109 ^a^ 71.4 ^b^	30 °C	[36]
1074	PEO/PA12	55	45	94 ^a^ 106 ^b^	30 °C	[36]
				110.7	25 °C	[37]
4033	PTMO/PA12	53	47	81 ^a^ 95 ^b^	30 °C	[36]

PEO: polyethylene oxide; PTMO: polytetramethylene oxide; PA6: polyamide 6; PA11: polyamide 11; PA12: polyamide 12; ^a^ prepared using 1-butanol as solvent; ^b^ prepared using cyclohexanol as solvent.

**Table 4:** Apparent activation energy for permeation (*E*p), evaluated in the temperature range of 15–55 °C, for Pebax^®^Rnew films and representative Pebax/ILs samples. *E*p1 below the IL *T*m; *E*p2 above the IL *T*m.

Sample		Activation Energy for Permeability (kJ mol^−1^)			
		CO_2_	O_2_	N_2_	H_2_
Neat Rnew	*E*p	17.0	28.0	32.7	26.7
Rnew–10% IL2	*E*p1	22.8	34.6	42.3	27.3
	*E*p2	38.0	50.4	53.6	41.4
Rnew–20% IL2	*E*p1	26.0	37.2	42.3	34.1
	*E*p2	47.6	55.3	62.2	45.3
Rnew–10% IL1	*E*p1	18.8	29.0	37.1	19.1
	*E*p2	19.9	33.9	40.6	30.2
Rnew–20% IL1	*E*p1	43.3	49.7	59.6	35.6
	*E*p2	30.5	42.9	46.6	46.3

## Data Availability

Data presented in this study are available on request from the corresponding author.
